# Whose rhythms? Rhythmic agency, rhythmic justice, and circadian health in the menopausal transition

**DOI:** 10.1177/17455057261491528

**Published:** 2026-09-20

**Authors:** Mary Ntochuwku Ndu, Ehinomen C. Oko-Oboh, Ailin Jalili, Charity Oga-Omenka

**Affiliations:** 1 School of Public Health Science, 339470 University of Waterloo, Waterloo, ON, Canada

**Keywords:** menopausal transition, circadian disruption, rhythmic agency, rhythmic justice, sleep health, social determinants of health; midlife

## Abstract

Sleep disturbance and circadian disruption affect 40–60% of women during the menopausal transition, yet public health guidance continues to frame circadian health primarily as a matter of individual behaviour. This commentary argues that such guidance assumes a degree of control over time, light, and rest that many women do not have. Drawing on chronobiology and feminist scholarship on time, we introduce rhythmic agency, the capacity to shape the environmental and temporal conditions that regulate circadian rhythms, as a framework for understanding why women frequently cannot act on behavioural framed advice in everyday life. We extend this with rhythmic justice, which examines how that capacity is unevenly distributed across caregiving roles, occupational demands, and built environments. Together, these concepts reposition circadian disruption during the menopausal transition as a problem of social organisation rather than individual regulation. We close by outlining research directions and policy levers, including work scheduling, nighttime communication norms, urban lighting, and caregiving support, that follow from treating circadian health as a socially distributed capacity.

## Introduction

Circadian rhythms regulate many aspects of women’s physiology, including sleep–wake cycles, metabolism, mood, and reproductive hormone secretion. These biological rhythms are synchronised by environmental cues, particularly light–dark cycles, which help coordinate daily patterns of activity and rest. Increasing evidence shows that disruptions to circadian rhythms can affect reproductive health, sleep quality, and metabolic regulation.^1,2^ Over the last recent decades, social environments have extended activity further into nighttime hours, weakening the environmental cues that normally synchronise circadian timing. Digital connectivity, shift work, and irregular schedules expose individuals to artificial light and delayed sleep timing, contributing to circadian misalignment.^2,3^

During the menopausal transition, hormonal changes can increase vulnerability to sleep disturbance and circadian disruption. This transition spans premenopause, the reproductive stage before noticeable hormonal change,^4^ and perimenopause, when irregular menstrual cycles and hormonal fluctuations precede the final menstrual period.^5,6^ Premenopause, by contrast, more broadly describes the reproductive stage before noticeable hormonal change.^4^ Sleep disturbance and circadian disruption are among the most commonly reported symptoms during this transition, affecting an estimated 40–60% of individuals.^7–9^ Longitudinal evidence from Study of Women Across the Nation (SWAN) also shows that insomnia symptoms were present in 31% to 42% of perimenopausal women at any one-year study interval, indicating that sleep disruption is not only common but persistent across the menopausal transition.^10^ Circadian factors, including nighttime light exposure and irregular sleep schedules, can compound these disruptions. Experimental circadian studies further suggest that postmenopausal women may experience weakened circadian signalling for sleep and wakefulness, with altered timing of sleep, alertness, and core body temperature rhythms.^11^

Circadian rhythms operate not only at the level of behaviour but also within reproductive physiology. Research has identified circadian clocks within ovarian tissue that regulate processes such as steroid hormone production, follicle development, and ovulation timing.^12,13^ These clocks rely on core circadian genes, which generate daily rhythms to coordinate reproductive hormone cycles. Other studies show that disrupting these circadian mechanisms can affect fertility and reproductive hormone regulation.^7,14^ Environmental signals, particularly light exposure play a critical role in synchronising these rhythms. Melatonin, which is released during darkness, helps coordinate circadian timing and supports ovarian function.^15^ These biological changes are also shaped by the hormonal transition itself. Reviews of menopausal sleep disturbance identify declining estrogen and progesterone, vasomotor symptoms, mood symptoms, and melatonin-related circadian changes as interacting pathways through which the transition can disrupt sleep continuity and circadian timing.^9,16,17^

Biological rhythms, however, do not operate independently of everyday life. They are continually shaped by the social organisation of time that structures when people wake, work, eat, and rest. Research in chronobiology and sleep science shows that circadian rhythms are shaped not only by internal physiology but also by environmental and social timing.^18^ In contemporary societies, sleep–wake patterns are increasingly structured by work schedules, digital communication, and economic activity rather than natural light–dark cycles.^2,19^ Social routines such as commuting, school schedules, and workplace hours can synchronise or disrupt circadian rhythms by shaping the timing of daily activities.^20^ Chronotype research adds another layer to this argument by showing that misalignment between internal circadian preference and external schedules is linked to poorer health outcomes in women. In a diverse sample of women, evening chronotype was associated with poorer cardiovascular health and adverse health behaviours even after adjustment for sociodemographic factors and menopausal status.^21^ Feminist health scholarship has long emphasised that women’s health cannot be understood apart from the social organisation of work, caregiving, and everyday life. Sociological research has similarly documented the gendered nature of sleep disruption among mid-life women, showing that women’s sleep is routinely subordinated to caregiving and household responsibilities.^22^

Public health responses to circadian disruption often emphasise behavioural recommendations such as improving sleep hygiene, reducing evening screen use, or maintaining consistent bedtimes.^23,24^ While these strategies can improve sleep for some individuals, they assume that individuals have sufficient control over their daily schedules and environments to follow such guidance. A study in Mexico City found that work demands, caregiving responsibilities, and constrained living conditions limit control over sleep timing and shape women’s sleep experiences.^25^ Similarly, another study using a socioecological lens described insomnia as embedded within social and societal conditions, including economic strain and household responsibilities.^26^ These findings indicate that circadian disruption is embedded within everyday social arrangements rather than arising solely from individual behavioural choices. They also point to a gap in how circadian disruption is currently conceptualised and suggest the need for frameworks that link circadian biology with the organisation of time in everyday life.

Existing research has established that sleep disturbance during the menopausal transition is common, biologically plausible, and linked to hormonal and circadian changes. This commentary does not take the position that circadian disruption is the sole explanation for menopausal sleep difficulties. Instead, it focuses on one underexamined dimension of this experience. We are, specifically, focused on the social conditions that shape whether women can act on circadian health advice and maintain circadian-supporting routines in everyday life. Behavioural guidance often places responsibility on individuals, treating difficulty following advice as a problem of knowledge, motivation, or effort. We instead focus on the temporal and environmental conditions that make such advice easier or harder to follow. From this perspective, difficulty following guidance may reflect the conditions in which people live and work rather than individual capability. We introduce rhythmic agency to capture this relationship. for examining how control over time, rest, and recovery influences the capacity to maintain circadian alignment. We recognise that multiple factors such as vasomotor symptoms, hormonal fluctuations, psychosocial stressors, and circadian processes shape sleep disturbance. It is, therefore, important to differentiate the following key concepts: sleep disturbance, circadian disruption, and circadian misalignment, we used in this commentary. Sleep disturbance refers broadly to difficulties initiating, maintaining, or restoring sleep.^27^ Circadian disruption refers to disturbances in endogenous circadian rhythms.^28,29^ Circadian misalignment describes a mismatch between biological timing and behavioural schedules.^30^ Although related, these concepts are not interchangeable and operate at different levels of analysis. Clarifying these concepts allows us to distinguish physiological circadian processes from the social and structural conditions that influence when individuals can align their daily activities with those processes. In this commentary, sleep disturbance is treated as a possible experienced outcome, circadian disruption as a disturbance in underlying biological rhythms, and circadian misalignment as the condition that may arise when biological timing conflicts with socially organised patterns of work, caregiving, light exposure, and rest. This distinction clarifies how rhythmic agency may shape circadian health without implying that circadian processes are the sole cause of menopausal sleep disturbance.

This commentary addresses two questions: 1) What structural conditions shape women’s capacity to maintain circadian alignment during the menopausal transition? 2) How do these conditions challenge behaviourally framed models of circadian health? Together, they position circadian disruption as a problem of social organisation rather than individual regulation. The following sections develop the concept of rhythmic agency, examine how domestic and institutional schedules shape circadian health, and consider the implications for rhythmic justice and public health policy.

## Rhythmic Agency

Rhythmic agency refers to the degree of control individuals have over the environmental and temporal conditions that regulate circadian rhythms, exposure to natural light and darkness, the timing of sleep and wakefulness, and opportunities for rest and recovery. It shifts attention from individual behaviour to the conditions that make such behaviour possible, linking circadian biology with the social organisation of time and highlighting how everyday environments enable or constrain the maintenance of biological rhythms.

Although related to temporal autonomy, schedule control, work-time flexibility, and time poverty, rhythmic agency is not synonymous with these concepts. Temporal autonomy generally refers to a person’s ability to determine how and when time is used, while schedule control and work-time flexibility focus more specifically on discretion over employment schedules. Time poverty describes the scarcity of discretionary time produced by competing demands. These concepts help explain who controls time and how time is allocated, but they do not necessarily examine whether available temporal and environmental conditions support biological synchronisation. Rhythmic agency is differentiated by its focus on the social and environmental conditions that support circadian alignment. It describes the socially conditioned capacity to shape or negotiate the timing and environmental conditions required for circadian alignment, including light and darkness, sleep and wake timing, temporal predictability, and opportunities for rest and recovery. A woman may therefore have some temporal autonomy, for example, flexibility over when she completes paid work, yet have limited rhythmic agency because nighttime caregiving, housing conditions, or expectations of constant digital availability prevent her from establishing circadian-supporting routines. In contrast, another woman may have relatively little discretion over working hours but still experience greater rhythmic agency if work schedules are predictable and the surrounding home and neighbourhood environments consistently support sleep and recovery. Rhythmic agency therefore does not replace existing concepts of temporal control. It identifies a particular pathway through which the organisation of time and environments becomes consequential for circadian health.

Rhythmic agency should not be understood as an individual trait, skill, or ability that some women possess more than others. Instead, it reflects the relationship between a person and the temporal and environmental conditions in which she lives. Two women with similar knowledge, motivation, and intentions may therefore have very different rhythmic agency if their circumstances differ. For example, a woman working a predictable shift with protected recovery time may have greater opportunity to maintain circadian-supporting routines than a woman who is regularly on call overnight. The difference lies not in the women themselves, but in what their circumstances make possible. This distinction also shifts how responsibility is understood. Limited rhythmic agency points to constraints in the organisation of time and environments rather than deficits in individual behaviour. Many of the conditions that shape rhythmic agency, including shift schedules, expectations of after-hours availability, lighting, and caregiving support, are determined by workplaces, institutions, communities, and governments. Individual guidance, such as sleep-hygiene advice, may still be useful, but the ability to follow that guidance depends on whether women have the conditions needed to do so. From this perspective, difficulty following circadian health recommendations may indicate constraints in the surrounding environment rather than a lack of knowledge, motivation, or effort. Figure 2 makes this visible: both dimensions of the matrix, are set by actors other than the individual woman, so movement between quadrants depends on institutional change rather than personal effort. Rhythmic agency therefore shifts attention from individual behaviour towards the social and institutional organisation of time.

The value of this reframing is both conceptual and practical. It identifies the conditions that need to change and where responsibility for those conditions lies. Each domain points to specific arrangements shaped by employers, institutions, or governments, such as predictable schedules, protected recovery time, expectations around after-hours communication, and lighting standards. This shifts the focus of intervention from changing individual knowledge or motivation to changing the conditions that make circadian health practices possible. It also changes how difficulty following health advice is understood. Rather than treating non-adherence as individual failure, rhythmic agency draws attention to whether women have the conditions needed to act on that advice. Behavioural approaches describe what women should do; rhythmic agency asks what needs to be in place for them to do it. Sociological work on rhythm analysis emphasises that individuals are not passive subjects of time but actively participate in shaping and negotiating everyday rhythms.^31^ From Lyon’s perspective, participation is not simply adaptation but can itself produce temporal misalignment. Women are continually reproducing, negotiating, and resisting the rhythms imposed by work, household, and built environments and in doing so generate the patterns of circadian misalignment called social jetlag.^32^ Social jetlag is a term Roenneberg coined to describe how the gap between endogenous biological timing and socially imposed schedules. Drawing on these perspectives, rhythmic agency highlights how the stability of biological rhythms depends partly on the temporal environments within which daily activities occur. Circadian health can therefore be understood as the outcome of an ongoing process of reconciling endogenous biological rhythms with exogenous social structures. This framing challenges models that treat circadian regulation as primarily a matter of individual compliance with behavioural guidelines.

Related research has examined how individuals negotiate control over institutional and technologically mediated rhythms.^33,34^ Studies using rhythm analysis show that workers often adapt and coordinate their bodily practices with institutional schedules, actively shaping the tempo of everyday activity rather than simply following externally imposed time structures.^35,36^ Extending these insights to circadian health highlights how temporal environments shape the conditions required for circadian regulation. These conditions include access to natural light and darkness, flexibility in sleep and wake times, the ability to disconnect from work or digital communication during the night, and opportunities to rest when fatigue signals the need for recovery. Women with higher rhythmic agency can organise their schedules in ways that support circadian alignment. Those with limited rhythmic agency must repeatedly override biological signals to meet external demands. Biological rhythms rely on consistent environmental signals. Rhythmic agency therefore provides a framework for understanding how variations in control over daily schedules and environments may influence sleep stability, hormonal regulation, and reproductive health.

Rhythmic agency is shaped by a range of structural conditions and varies across individuals. Work schedules, caregiving responsibilities, digital connectivity, and patterns of artificial light exposure can all influence individuals’ ability to maintain circadian-supporting environments. Traditional sleep research often measures outcomes such as sleep duration or bedtime consistency. While these metrics are informative, they do not capture the social and environmental conditions that constrain sleep behaviour. Women’s daily routines frequently involve balancing paid employment with unpaid caregiving and household responsibilities. This balancing reduces control over sleep timing and opportunities for recovery. Sociological research has described this condition as time scarcity, where limited control over time constrains opportunities for rest.^37,38^

During the menopausal transition, coordinating biological rhythms with social demands becomes more difficult. Hormonal fluctuations may heighten sensitivity to circadian disruption, so stable rhythms are harder to maintain when environmental conditions are inconsistent. Rhythmic agency therefore helps explain how the effortful process of coordinating biological rhythms with social expectations may contribute to sleep disturbance and hormonal instability during this transition. The four domains bring together insights from chronobiology and social science research on the organisation of time. Chronobiology identifies the conditions that support circadian alignment. Social science research shows how those conditions are shaped by work, caregiving, digital expectations, and the environments in which people live. Bringing these perspectives together, we identify four domains: occupational time, domestic and caregiving time, digital time, and the light environment. These domains describe socially organised conditions that shape opportunities for circadian alignment. Individual factors such as chronotype play a different role. They may influence how a person responds to these conditions without determining the conditions themselves. We therefore treat them as moderators. For example, a work schedule may determine when sleep is possible, while chronotype may influence how disruptive that schedule is.

We identified the four domains through a conceptual synthesis rather than primary data collection. We first considered the temporal and environmental conditions that chronobiology identifies as important for circadian alignment. We then drew on feminist and sociological research on the organisation of time, alongside qualitative research on women’s sleep at midlife, to examine the social arrangements that shape these conditions. Recurring arrangements were grouped according to how they influence circadian alignment. We retained domains that were socially or institutionally organised, appeared across the relevant literatures, and operated through a distinct pathway. We also considered commuting and transport time, meal timing, and neighbourhood noise. These conditions did not operate through a distinct pathway, so we treated them within the existing domains rather than as separate domains. The resulting four domains are intended as an initial conceptual structure rather than an exhaustive framework and remain open to refinement through empirical research.

The concept of rhythmic agency requires further empirical development. As an initial framework, rhythmic agency may be understood as the capacity to maintain circadian-supporting conditions across the four interconnected domains presented in Table 1.

**Table 1.** Conceptual domains and potential illustrative indicators of Rhythmic Agency.

Policy Domains	Structural Time Conditions	Rhythmic Agency Domain	Potential measurements	Potential Circadian Outcomes
Occupational health policy	shift work, unpredictable schedule	Work schedule flexibility, recovery opportunities,	Ability to modify work hours; access to recovery time; schedule stability	Sleep-wake timing, circadian alignment, sleep efficiency
Organisational and digital policy	Digital connectivity, after-hours communication, constant availability expectations	Digital autonomy, temporal boundary control	Ability to disconnect; freedom from after-hours communication expectations	Melatonin secretion, sleep onset timing, sleep continuity
Urban planning and public health policy	Artificial light exposure, neighbourhood lightscapes, built environment conditions	Light environment control, environmental rhythm support	Ability to regulate light exposure; access to circadian-supportive environments	Circadian entrainment, melatonin regulation, sleep quality
Social policy	Caregiving responsibilities, domestic labour, household temporal demands	Sleep timing control, household temporal autonomy	Ability to determine bedtime and wake time; ability to protect time for recovery	Circadian stability, recovery capacity, reproductive and metabolic health

These domains are intended to guide future conceptual and measurement development and do not represent a validated instrument.

The four domains do not operate independently or simply add together. Occupational time and domestic or caregiving time are the main structuring domains as they determine when sleep and recovery are possible. Their effects can also compound overtime. For example, an early work shift may be manageable on its own but become much more disruptive when combined with overnight caregiving, as illustrated in Figure 2. Digital availability and the light environment on the other hand play a different role. Instead of determining when a woman must be awake, they can affect the quality of the time available for sleep and recovery. After-hours messages or light entering the bedroom, for example, may not reduce the amount of time available for sleep, but they can make that time less supportive of circadian alignment. The domains are therefore unlikely to have equal effects. We expect occupational and domestic or caregiving constraints to have the greatest influence because they shape the time available for sleep and recovery. Digital and light-related conditions may become particularly important when that time is already limited. The domains may also overlap in practice. Women working non-standard schedules, for example, may also face caregiving demands or housing conditions that make recovery more difficult. These relationships have implications for both research and intervention.

The relative importance of each domain may also change across the life course. Occupational time and digital demands may be particularly important in early adulthood, while domestic and caregiving responsibilities may become more influential during periods of intensive childrearing. These pressures may overlap in midlife. In later life, environmental and institutional conditions may become more important, particularly in care settings where lighting, mealtimes, and daily routines are organised by others. The contribution of each domain should therefore be examined across different life stages rather than assumed to be the same throughout the life course. Figure 1 illustrates the pathway of rhythmic agency and how it mediates the relationship between social time structures and circadian health. Figure 2 extends this model by illustrating how circadian pressure interacts with rhythmic agency. Circadian pressure refers to external demands that disrupt biological rhythms, such as shift work, nighttime caregiving, or constant digital connectivity. When circadian pressure is high and rhythmic agency is limited, the risk of circadian misalignment increases. The matrix is organised around the two primary domains. Digital availability and the light environment are included within the quadrant profiles and not treated as separate axes because they influence how supportive the available time is for sleep, recovery, and circadian alignment.

**Figure 1.**
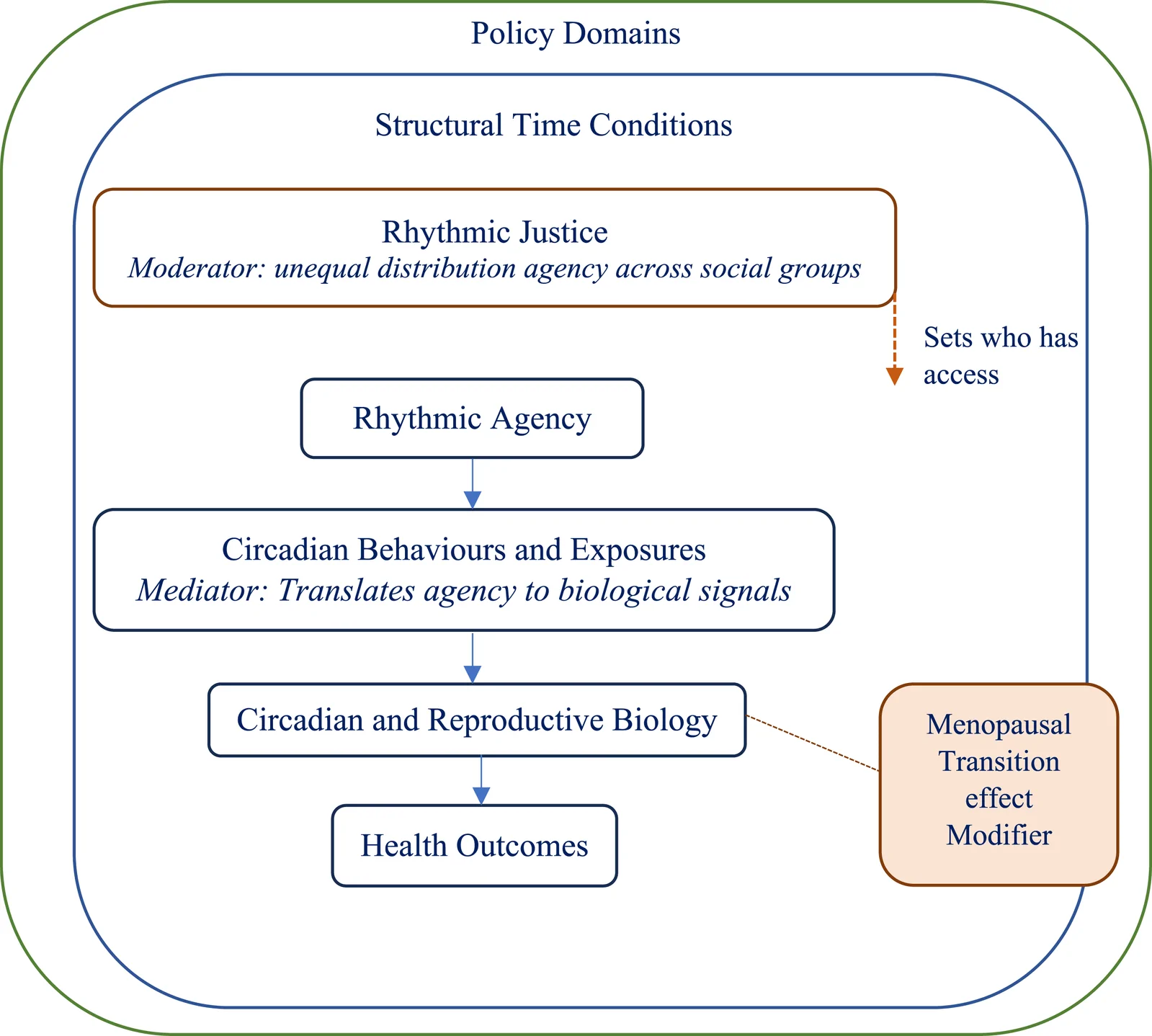
Rhythmic agency, a model linking social time and women’s circadian health.

**Figure 2.**
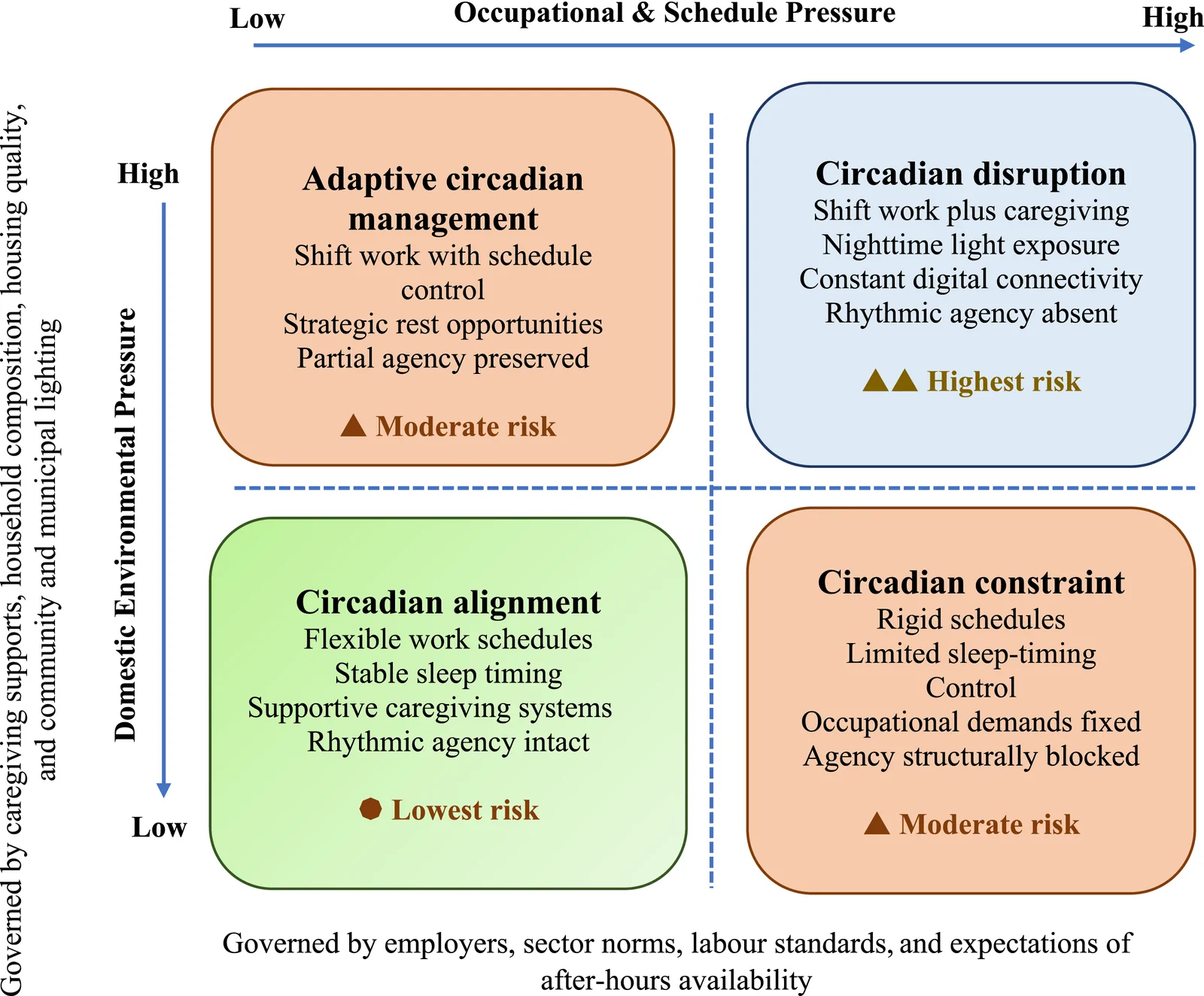
Circadian pressure matrix: four experiential profiles of rhythmic agency across occupational and schedule pressure and domestic environmental pressure. Both dimensions are structurally determined rather than individually chosen. **Adaptive circadian management:** Occupational pressure is present but partly offset by schedule control. Strategic rest opportunities preserve partial rhythmic agency; moderate risk. **Circadian alignment:** Both pressures are low. Flexible schedules and supportive systems preserve full rhythmic agency. **Circadian constraint:** High occupational pressure, low domestic. Rigid schedules block sleep timing autonomy; agency structurally is limited. **Circadian disruption:** Both pressures are high, compounding demands eliminate rhythmic agency; highest circadian health risk.

## Domestic and Institutional Schedules

Circadian disruption is often discussed in relation to work schedules, yet the home environment also shapes circadian rhythms. Household routines are structured by caregiving, communication, and domestic tasks that frequently extend into nighttime hours. In many households, women remain responsible for responding to children during the night, coordinating family schedules, and managing digital communication.^39^ These activities often involve exposure to artificial lighting and screens late in the evening. Such conditions can delay circadian timing and suppress melatonin production, making it harder to initiate or maintain sleep. The home environment, therefore, has its own lightscape, a pattern of illumination and activity that shapes circadian rhythms. These domestic environments are central to the distribution of rhythmic agency, because they shape the degree of control individuals have over light exposure and rest. Although homes are typically considered spaces of rest, they may also become environments where sleep is fragmented by caregiving demands and digital connectivity. Research during the COVID-19 pandemic showed how disruptions to everyday routines altered circadian patterns. Many women reported increased caregiving responsibilities and blurred boundaries between work and home life, contributing to sleep disturbance and irregular daily rhythms.^40^ These findings illustrate how circadian health is closely tied to the organisation of daily life.

Beyond the household, institutional schedules also shape circadian health. Modern labour systems often require work to be performed at times that conflict with biological rhythms. Shift work, early morning schedules, and expectations of constant availability can all disrupt circadian alignment. Research has linked shift work to changes in reproductive hormone levels, menstrual irregularities, and fertility challenges.^41^ These findings indicate that institutional time structures can affect reproductive physiology. Women balancing employment and caregiving responsibilities may experience ongoing tension between biological signals of fatigue and external expectations for productivity. In these situations, rest is often postponed to meet social obligations. Over time, repeated disruption of sleep and circadian rhythms may accumulate as chronic fatigue, insomnia, or metabolic strain. These outcomes highlight the need to consider how institutional schedules interact with biological rhythms. They also illustrate how limited control over institutional time structures can constrain individuals’ capacity to maintain circadian alignment.

Both axes are shaped by wider social and institutional conditions rather than individual choice. Work and schedule pressures are influenced by employers, workplace norms, labour standards, and expectations of after-hours availability. Domestic pressures are shaped by caregiving responsibilities and supports, household circumstances, housing conditions, and community lighting. A woman’s position in the matrix therefore reflects the conditions around her. Moving between quadrants depends largely on changes to these conditions, rather than greater individual effort.

## Rhythmic Justice and Circadian Health

In this section, we argue that women differ in their capacity to maintain circadian-supporting environments. Rhythmic justice asks the question: how and why is that capacity distributed unevenly across society, and what obligations do institutions have to address those inequalities? If circadian health depends partly on rhythmic agency, then the ability to maintain stable biological rhythms is shaped not only by individual behaviour but also by broader social conditions. Feminist scholarship on time has long demonstrated that the organisation of work, caregiving, and domestic life distributes temporal resources unevenly across populations.^37^ These inequalities shape the degree of control individuals have over the conditions that sustain biological rhythms. In this way, inequalities in rhythmic agency translate into inequalities in circadian health. From a chronofeminist perspective, public health approaches should support social conditions that enable rest, darkness, and temporal flexibility.^42^ Chronofeminist scholarship examines how gendered power relations shape the organisation of time. These conditions are unevenly distributed across populations, with opportunities to maintain circadian-supporting environments also distributed unequally.^37,43–47^ From this perspective, the concept of rhythmic justice extends the framework of rhythmic agency by examining how the capacity to maintain circadian-supporting environments is distributed across society. This reinforces the need to understand circadian health as both a biological process and a socially distributed capacity.

Rhythmic justice builds on, but is narrower than, broader accounts of temporal justice and theories of social justice.^37^ Temporal justice is concerned with how time, temporal autonomy, waiting, speed, and the burdens of coordinating everyday life are distributed across social groups 44). Rhythmic justice applies these concerns specifically to the conditions required for biological synchronisation. It asks who has access to predictable schedules, restorative time, darkness, daylight exposure, temporal boundaries, and the ability to respond to bodily signals of fatigue and recovery.

Rhythmic justice is also related to, but distinct from, recent work on circadian justice and sleep-health equity. Circadian justice considers how the benefits and burdens of circadian time are distributed as a matter of political justice.^43^ Sleep-health equity frames sleep as a modifiable public health resource that is unequally available across populations.^44^ Rhythmic justice differs from both in its focus on the environmental and temporal conditions required for biological synchronisation, rather than on sleep outcomes or the distribution of time alone. It asks not only who sleeps well, but who can access the light, darkness, predictability, and recovery on which circadian rhythms depend.

The distinctive concern of rhythmic justice is therefore not simply whether people have equal amounts of time. Rather, it is whether social institutions distribute the temporal and environmental conditions needed to sustain biological rhythms equitably. Equal discretionary time may not produce equal rhythmic agency when women experience different caregiving demands, occupational schedules, housing conditions, or exposure to nighttime light and noise.^37^ Rhythmic justice thus provides a normative framework for evaluating how labour systems, households, digital infrastructures, and built environments distribute opportunities for circadian alignment and risks of circadian disruption. It requires attention to how access to circadian-supporting conditions is distributed across social groups. Rhythmic agency is socially patterned by socioeconomic position, race and ethnicity, migration status, disability, occupational class, housing conditions, and age or life stage. These groups therefore experience unequal opportunities for circadian alignment. Rhythmic justice asks whether institutions distribute circadian-supporting conditions fairly, and what obligations they have to reduce these inequities. Integrative reviews of the menopausal transition similarly identify socioeconomic status, education, and employment as social determinants that shape women’s health at midlife.^48^ Women experiencing poverty may have limited control over work schedules, housing environments, or neighbourhood noise and lighting conditions. Migrant workers and racialised populations are disproportionately represented in occupations characterised by non-standard working hours. Individuals living with disabilities or chronic illness may face additional constraints on recovery and sleep timing. Urban and rural environments may also produce different forms of circadian vulnerability through transportation systems, employment opportunities, and access to services.

From this perspective, rhythmic justice is concerned not only with whether circadian health is achievable, but with who has access to the social conditions that make circadian health possible. Inequalities in temporal resources therefore become inequalities in biological opportunity. This perspective aligns with broader public health efforts to address health inequities through structural rather than solely behavioural interventions.

When access to circadian-supporting conditions varies across populations, circadian health becomes a matter of social distribution as well as physiology. Rhythmic justice therefore highlights how labour conditions, caregiving structures, built environments, and digital infrastructures shape the rhythms of everyday life. Framed in this way, circadian disruption during the menopausal transition is not only a clinical concern but an indicator of how temporal resources and supporting conditions are distributed across society. Women who carry disproportionate caregiving loads, work non-standard hours, or live in environments with limited control over light and noise face compounding constraints on rhythmic agency. These constraints may intensify during menopausal transition, when sleep disturbance sensitivity to circadian disruption may increase due to intersecting biological, social, and psychological factors. Addressing these inequalities requires moving beyond individual sleep advice to consider the structural conditions that shape whose rhythms can be sustained or distributed. Recognising these inequities raises important questions about how systems organise time, work, caregiving and environmental exposures. If organisation of work, caregiving, and build environment shape rhythmic agency, then interventions must target the structural conditions that either support or constrain circadian alignment. This approach shifts interventions away from focusing on women adapting to changes in the environment to examining the role of systems on rest and circadian alignment. Achieving rhythmic justice requires coordinated action across multiple sectors, including employers, public health agencies, health systems, and governments. The following policy domains illustrate how structural interventions across these sectors could strengthen rhythmic agency and support circadian health.

### Domain 1: Occupational health policies

The workplace is an important site for promoting rhythmic justice, particularly for women during the menopausal transition. Policies that promote predictable work schedules, adequate recovery periods following night shifts, forward-rotating shift patterns, menopause-responsive workplace accommodations, and flexible start and finish times where feasible may reduce circadian strain. Occupational health guidance could also encourage employers to minimise consecutive night shifts and incorporate fatigue management into workplace wellness programmes. Such measures recognise that labour systems shape exposure to circadian disruption and influence workers’ opportunities for recovery.

### Domain 2: Organisational and digital policies

Digital technologies are increasingly extending work into evening and nighttime hours. Organisational policies could establish protected right-to-disconnect periods. Specifically, organizations could delay non-urgent emails sent outside working hours, limit expectations for after-hours messaging, and encourage asynchronous rather than immediate communication where appropriate. Employers could also implement digital wellbeing policies that reduce unnecessary nighttime notifications and protect uninterrupted recovery time. These measures strengthen rhythmic agency by safeguarding temporal boundaries that support circadian alignment.

### Domain 3: Urban planning and public health policies

Built environments also shape circadian health. Urban lighting policies that reduce excessive nighttime illumination through shielded street lighting, adaptive lighting systems, and restrictions on unnecessary commercial light emissions may help preserve natural light-dark cycles. Neighbourhood design that increases access to natural daylight through walkable communities, green spaces, and building designs that maximise daylight exposure may further support circadian alignment. Public health agencies and municipalities could also incorporate circadian health into environmental health assessments, residential planning, transportation systems, and lighting standards for hospitals and long-term care facilities. Although the built environment is well recognised as a social determinant of health, its circadian implications remain underexplored in urban planning and public health policy.

### Domain 4: Social policies

Social policies that reduce caregiving burdens and increase temporal flexibility may also promote rhythmic justice. Paid family leave, affordable childcare, community-based eldercare, respite care services, home support programmes, and flexible caregiving leave can redistribute the temporal labour that disproportionately affects women during midlife. These interventions recognise that opportunities for rest, recovery, and biological synchronisation are partly determined by social policy choices rather than individual behaviour alone.

## Limitations

We need to acknowledge several limitations of our proposed framework. First, rhythmic agency and rhythmic justice are conceptual constructs that have not yet been validated empirically. Second, evidence linking social conditions to circadian outcomes remains heterogeneous and is drawn from multiple disciplines using different methodological approaches. Relatedly, the synthesis that produced the four domains was narrative rather than systematic, so a systematic review or a primary qualitative study could revise the domain structure. Third, while menopause provides an important entry point for examining circadian vulnerability, the framework may also be relevant to other populations experiencing circadian disruption and requires testing beyond the context of women’s health. Finally, the policy implications proposed here remain conceptual and also require empirical evaluation. The relationships proposed here may not operate in the same way across all stages of life. Rhythmic justice, as a principle concerned with how institutions distribute conditions that support circadian health, applies across the life course. However, the conditions that shape rhythmic agency, and their implications for health, may change with age and life stage. Reproductive and hormonal outcomes may be particularly relevant during the reproductive years and midlife, while cardiometabolic and cognitive outcomes may become more prominent later in life. The framework therefore provides a common structure while allowing the relative importance of its domains and health outcomes to vary across the life course. Future research comparing different life stages could help clarify these differences.

## Conclusion

This commentary has introduced rhythmic agency and rhythmic justice as a framework for understanding circadian health during the menopausal transition as both an individual capacity and a socially distributed condition. Reframing circadian disruption in this way moves the conversation beyond individual behavioural advice toward the conditions, work demands, caregiving responsibilities, digital pressures, and built environments, that shape the timing of everyday life. Four empirical priorities follow from this framework. First, develop and validate a scale to measure rhythmic agency that is different from existing sleep-hygiene and chronotype instruments. Second, test whether rhythmic agency mediates the association between caregiving load, occupational schedule, and circadian outcomes during the menopausal transition, and test whether it does so in the same way at other life stages. Third, examine whether interventions that increase rhythmic agency improve circadian and metabolic outcomes more than behaviourally framed sleep-hygiene interventions alone. Fourth, map the social distribution of rhythmic agency to identify where rhythmic injustice concentrates and where policy levers would have the greatest effect. Public health approaches, in turn, should support environments that enable rest, darkness, and temporal flexibility, particularly for women balancing paid work and caregiving roles. More broadly, the concepts of rhythmic agency and rhythmic justice invite researchers, practitioners, and policymakers to reconsider circadian health not simply as a matter of individual behaviour or biological vulnerability, but as a reflection of how societies organise time itself. Addressing circadian health therefore requires attention not only to bodies and behaviours, but also to the social conditions that determine whose rhythms can be sustained and whose are routinely disrupted.

## Data Availability

Not applicable. No datasets were generated or analysed for this commentary.*
